# Insights into the mechanical properties of a silicone oil gel with a ‘latent’ gelator, 1-octadecylamine, and CO_2_ as an ‘activator’

**DOI:** 10.3762/bjoc.6.111

**Published:** 2010-10-15

**Authors:** Emiliano Carretti, Mathew George, Richard G Weiss

**Affiliations:** 1 Department of Chemistry & CSGI Consortium, University of Florence, via della Lastruccia, 3, I-50019 Sesto Fiorentino (Florence), Italy; 2 Department of Chemistry, Georgetown University, Washington, DC 20057-1227, USA; 3 Sepax Technologies, Inc., Newark, DE 19711, USA

**Keywords:** ammonium carbamate, damping factor, gel-sol transition, rheology, viscosity

## Abstract

A detailed study of the rheological properties of silicone oil gels, made from a low-molecular-mass organic gelator, a combination of 1-octadecylamine (a latent gelator) and carbon dioxide (an ‘activating’ molecule), is reported. Information gleaned from the mechanical measurements is used to characterize the gel networks and how they respond to temperature and strain. It is shown, for example, that very precise measurements of the gel-to-sol transitions can be obtained from plots of viscosity versus temperature.

## Introduction

During the last two decades, research efforts have increased enormously to understand the range of structures and processes of self-assembly of ‘small’ molecules such as low-molecular-mass organogelators (LMOGs), which gelate large volume fractions of a liquid. The structures of the assemblies almost always consist of one-dimensional objects (usually fibers, rods or nanotubes) that interact to form three-dimensional self-assembled fibrillar networks (SAFINs) [1–3]. This interest is motivated by the search for fundamental information on the basis for anisotropic self-assembly and recognition of the potential applications for such gels [4–6] – most can be reverted thermally to their solution (or sol) states – as models for several important biological aggregates (e.g., that are involved with neurodegenerative and other diseases, such as Alzheimer’s, mad cow disease, and sickle cell anemia) that are much more complex structurally. Previously, we described simple methods by which ‘latent’ LMOGs (i.e., molecules that do not aggregate into SAFINs efficiently, or at all, unless a chemical stimulant, such as an acid [7] or a triatomic gas [8–9], especially carbon dioxide [10–11], is added).

Although the structures of SAFINs and rheological properties of their gels made with ‘normal’ LMOGs have been extensively investigated [2,12], the mechanical properties of gels made from latent LMOGs have not received much attention because their rheological measurements are fraught with experimental difficulties. Many of the gels from latent LMOGs are mechanically fragile and difficult to make in a manner that ensures a reasonable degree of reproducibility. Here, we describe a detailed rheological study of gels made from a latent LMOG, 1-octadecylamine (**ODA**), and a triatomic molecular ‘activator’, carbon dioxide, with silicone oil, tetramethyltetraphenyltrisiloxane, as the liquid component [10–11]. This liquid has been selected because its very low vapor pressure avoids a potential complication, i.e., evaporation of a portion of the liquid during long-term measurements.

## Results and Discussion

**Scheme 1:**
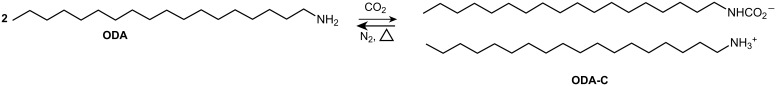
Reversible reaction of octadecylamine with carbon dioxide.

**General considerations.** In previous work, we have reported the properties of these gels and others made with a variety of amine latent LMOGs, other triatomic gases, and a wide range of liquids [8–11]. The basic process to form the gels is simple: bubbling CO_2_ through a solution of the amine and the liquid for a minute or longer (to ensure complete reaction). The CO_2_ adds to one amino functionality, forming a carbamate, while a proton of the amine is transferred to a second amine molecule (Scheme 1) [13]. The resultant ammonium carbamate from **ODA**, C_18_H_37_NHCO_2_^− +^H_3_NC_18_H_37_ (**ODA-C**), is held together much more strongly, by electrostatic forces, than uncharged latent LMOG molecules. Especially in liquids of low polarity, the electrostatic forces and ion pairing are very strong and they are the basis for the induced self-assembly leading to one-dimensional objects and SAFINs. The concentrations of the latent LMOG can be very low, <1 wt %. For the purposes of this work, somewhat higher concentrations were employed to ensure that the rheological measurements probe viscoelasticity and not simple Newtonian viscosity effects of the liquid.

It is known that the fibers constituting the SAFINs of the **ODA-C**/silicone oil gels are crystalline, and that the molecules of **ODA-C** are in extended conformations in lamellae with their long axes normal to the lamellar planes [10]. In addition, molecules of silicone oil are not inside the fibers [14] because the powder diffraction patterns of the neat **ODA-C** powder and the SAFIN structures in the gel are the same (i.e., the **ODA-C** molecules pack in the same morph in the absence and presence of silicone oil).

**Figure 1:**
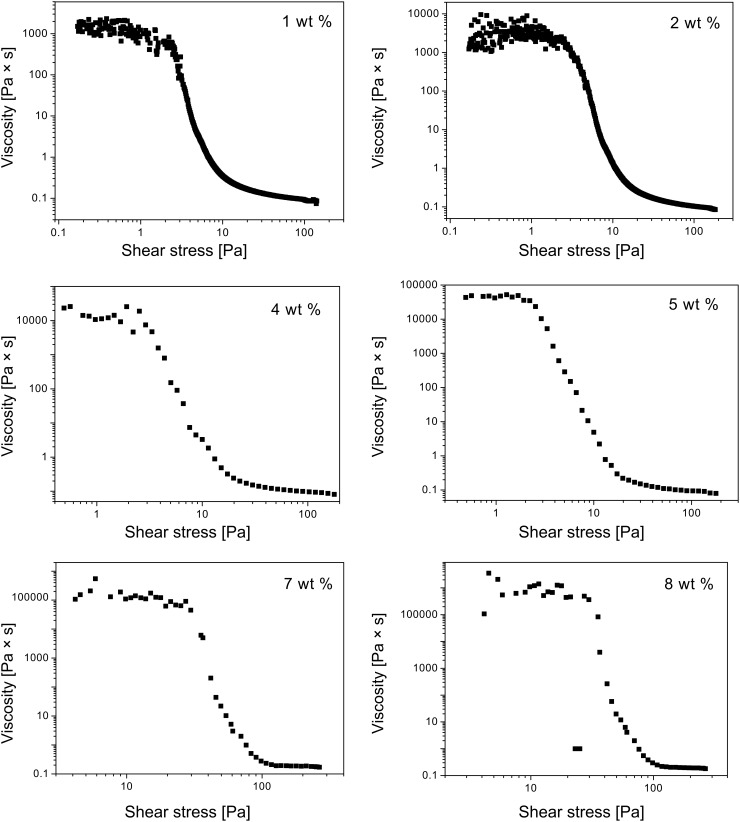
Flow curves of **ODA-C**/silicone oil gels at 25 °C and increasing stress.

**Figure 2:**
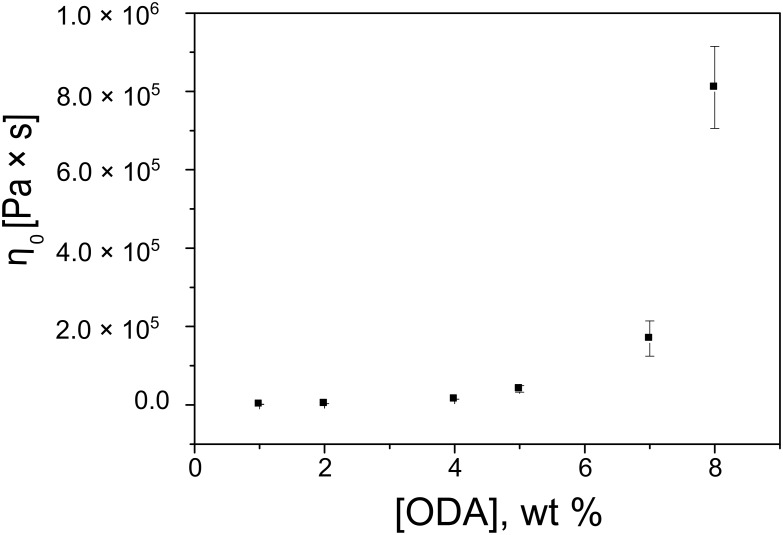
*η*_0_ values as a function of **ODA** concentration.

**Flow curves.** The rheological behavior of the gels was explored over a torque range from 5 × 10^−4^ to 100 mN m while increasing the torque logarithmically. This procedure ensures that the same protocol for increasing torque is applied to all the samples. Figure 1 shows the flow curves of **ODA-C**/silicone oil gels made from 1, 2, 4, 5, 7, and 8 wt % **ODA**. The shapes are similar and typical of viscoelastic shear thinning materials. The ‘lower Newtonian region’, indicated as *η*_0_, corresponds to the value of the horizontal asymptote (Table 1) and is plotted as a function of **ODA** concentration in Figure 2.

**Table 1:** Values of *η*_0_ from Figure 1 and *T*_g_^a^ from Figure 3. The activation energies (*E*_a_)^b^ have been calculated from Arrhenius fits of the data in Figure 3, plotting ln(*η*) versus 1/*T* for points above and below *T*_g_. The numbers in parentheses are the *E*_a_ values normalized for LMOG content by dividing the gross numbers by the weight fraction of **ODA**.

Gelator wt %	*η*_0_ (Pa·s)	*T*_g_ (°C)	*E*_a_ above *T*_g_ (kJ mol^−1^)	*E*_a_ below *T*_g_ (J mol^−1^)

1	1330 ± 430	43.0 (48)	0.10 (0.001)	0.091 (9.1 × 10^−4^)
2	3290 ± 50	50.4 (59–60)	5.01 (0.025)	38.9 (0.20)
4	14700 ± 100	64.2 (79)	12.0 (0.030)	919 (2.3)
5	40800 ± 8300	70.3 (80)	21.5 (0.043)	961 (1.9)
7	169000 ± 45000	74.5	42.6 (0.061)	520 (0.74)
8	810000 ± 105000	82.3	24.9 (0.031)	529 (0.66)

^a^Values in parentheses from the falling drop method [11]. ^b^The numbers in parentheses are the *E*_a_ values normalized for LMOG content by dividing the gross numbers by the weight fraction of **ODA**.

**Temperature effects***.* In our previous study with these gels [10], the values of the sol–gel transition temperatures (*T*_g_) were measured by the ‘falling drop’ method [2,15–16]. This crude test has become a standard method, although it does not yield a value that is reproducible between laboratories – the thickness of the gel sample, the diameter of the vial, and the surface of the vial are variables that can alter the *T*_g_ measured in this way. By contrast, rheological measurements, when conducted in the linear viscoelastic regions, provide *T*_g_ values that should be reproducible in any laboratory provided the samples are prepared in the same way. However, it must be recognized that because the gels are under some shear stress, their *T*_g_ values may be slightly affected. In the experiments conducted here, the mechanical effects of shear on *T*_g_ are expected to be very small because the stress applied to the samples is in the lower Newtonian region, where the dependence of the viscosity on applied shear stress is minimized and the gelator network structures (i.e., self-assembled fibrillar networks or SAFINs) are pre-established to avoid shear alignment. Also, the very low solubility of the ammonium carbamate salt in silicone oil indicates that a very high fraction of **ODA-C** remains part of the SAFIN structure even at elevated temperatures that approach *T*_g_ [13].

**Figure 3:**
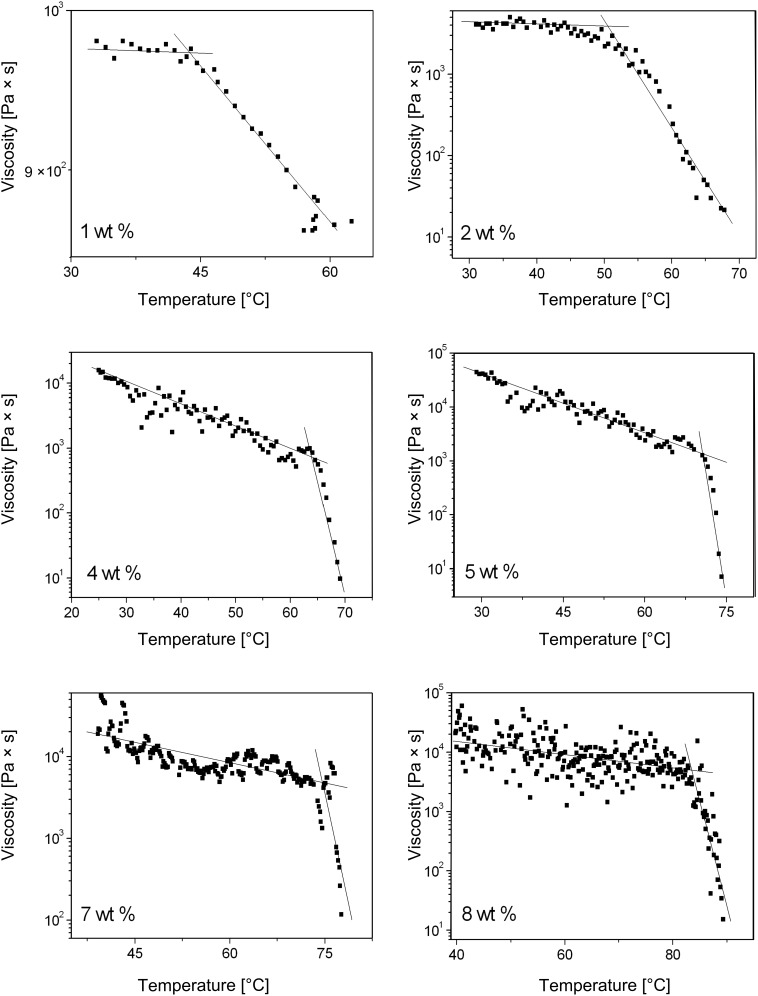
Dependence of viscosity of **ODA-C**/silicone oil gels on temperature at a shear stress of 1 Pa. From top left to bottom right: [**ODA**] = 1, 2, 4, 5, 7, and 8 wt %. Note that the viscosities of the 1 wt % sample have been plotted linearly and those at the other concentrations are plotted semi-logarithmically.

From the intersection between the straight lines drawn from the linear portions of the data points in Figure 3, very precise values of *T*_g_ can be determined at each gelator concentration. Note that, as expected, the viscosities decrease much more precipitously upon increasing temperature above *T*_g_. On comparison, the *T*_g_ values obtained rheologically and by the falling drop method [11] follow the same trend, but those from the data in Figure 3 are always lower (Table 1). Regardless, those values determined rheologically are closer to instrumentally independent parameters because, unlike the values from the falling drop method (which depend on the diameter of the container and the height of the gel) [16], they do not depend as acutely on the geometry of contact with the sample holder.

All viscosities were fit reasonably to the Arrhenius model to obtain activation energies for viscous flow. Such energies of very complex fluids (e.g., honey) are usually expressed per mole [17] and we have done so here. However, it is probably not correct to do so for a micro-separated 2-phase system such as a gel. Even with our careful attempts to prepare the gels so that they can be made reproducibly, the activation energy data indicate that we have only been partially successful. The rough trends in the activation energies indicate that the SAFINs of the gels differ at the microscopic level for reasons other than differing LMOG concentrations; there are inherent inhomogeneities that are a result of stochastic nucleation and microscopic growth effects. Although two gels made by the same protocol from aliquots of the same sol may appear to be the same macroscopically, they must differ in important ways on small length scales. Regardless, it is clear that the activation energies associated with fluidity changes increase as the **ODA** concentration increases; the effect is not linear. Furthermore, the influence of the SAFIN on viscous flow is apparent in the >100-fold higher activation energies in the gel phases than in the corresponding sol phases. Thus, more macroscopic measures of the gel properties, such as *T*_g_, do follow smooth trends with LMOG concentration, and the *T*_g_ values, especially as measured by the falling drop method [2], are much more reproducible than the absolute values of the viscosities.

**Figure 4:**
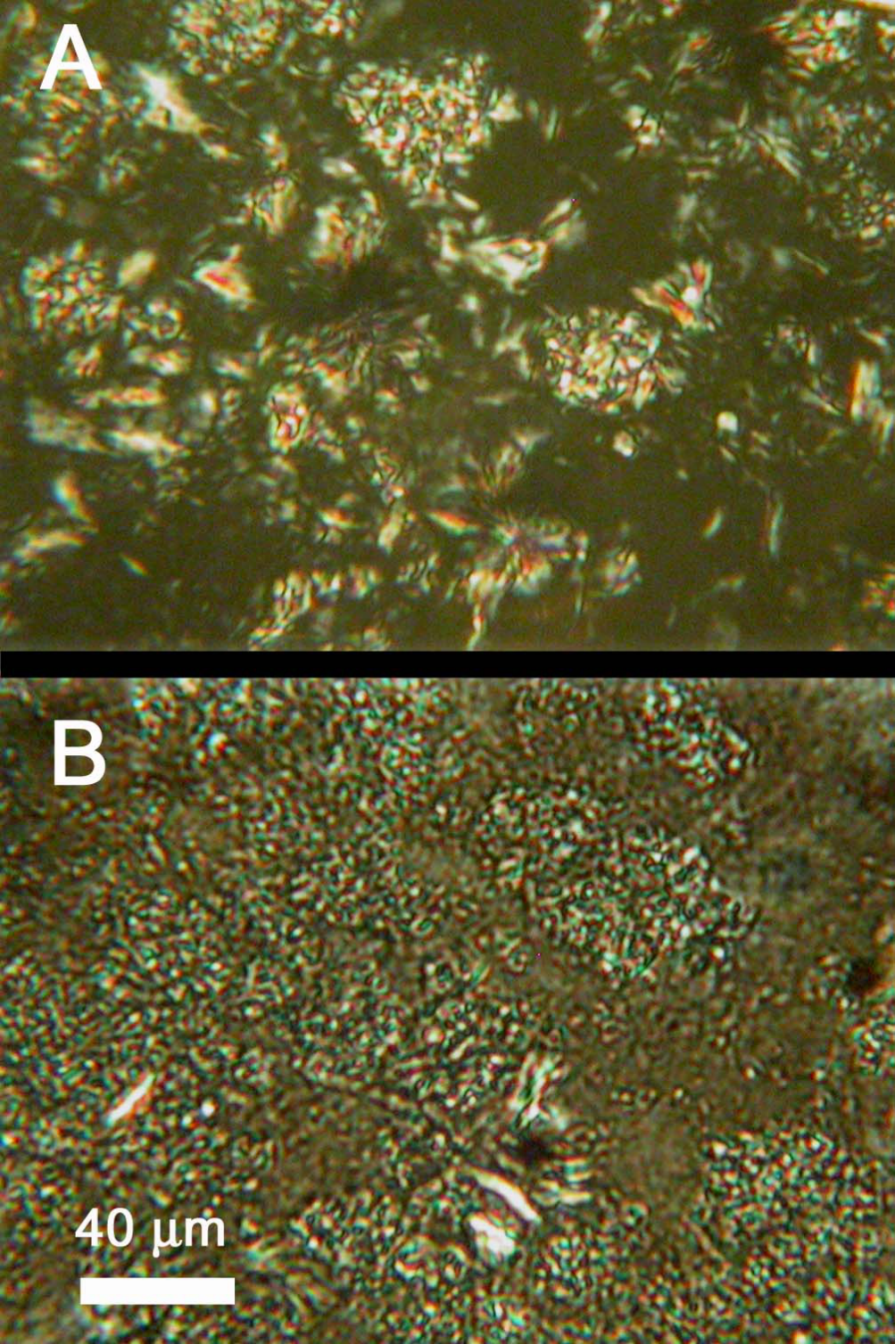
Optical micrographs of a 2 wt % **ODA-C**/silicone oil gel before (A) and after (B) the application of 10 Pa shear stress for 10 min at 25 °C. The space bar applies to both micrographs.

**Optical microscopy.** Optical micrographs of 2 wt % gel samples were recorded before and after the application of a constant and continuous stress of 10 Pa for 10 min. The micrograph in Figure 4A is typical of those previously reported [11]. As can be seen, aggregates before the application of the stress are more elongated and fiber-like than those after the perturbation (Figure 4B). The changes can be attributed to the partial breaking of the SAFIN that occurs when a large stress is applied to the system. Although the network is ‘damaged’, it is not destroyed even after this treatment: the viscosity of the perturbed sample remains significantly higher than that of neat silicone oil. In fact, the appearance of the gel in Figure 4B is very similar to that reported for an **ODA**/silicone oil gel (i.e., in the absence of CO_2_) [11].

The changes are attributed to irreversible breaking of fibers as well as cleavage of some of their junction zones. Unlike non-crystalline SAFINs, such as those from giant worm-like micelles, only in exceptional cases are crystalline SAFINs able to ‘heal’ without being melted (i.e., transformed into the sol phase) and then reformed by cooling.

**Figure 5:**
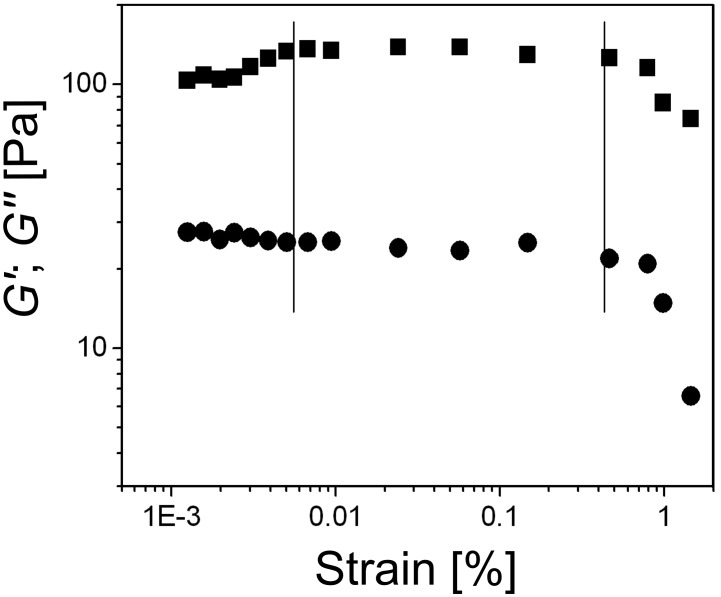
Amplitude sweep test of a 2 wt % **ODA-C**/silicone oil gel showing *G*′ (■) and *G*″ (●). The vertical lines mark the range of the linear viscoelastic region.

**Amplitude sweep.** The stability of the structures of viscoelastic substances is determined by their response to increasing strain and is measured as the limit of the linear viscoelastic strain range, the point where both *G*′ and *G*″ values begin to decrease with increasing strain; usually, *G*′ begins to decrease at lower strain values compared to *G*″. Beyond the strain limit, the SAFIN is damaged and eventually destroyed. Thus, the behavior of *G*′ and *G*″ as a function of the angular frequency must be determined at a strain value within the linear viscoelastic range. For the amplitude sweep experiments shown in Figure 5, a 2 wt % **ODA-C**/silicone oil gel and a 1 Hz frequency of oscillation were chosen.

**Figure 6:**
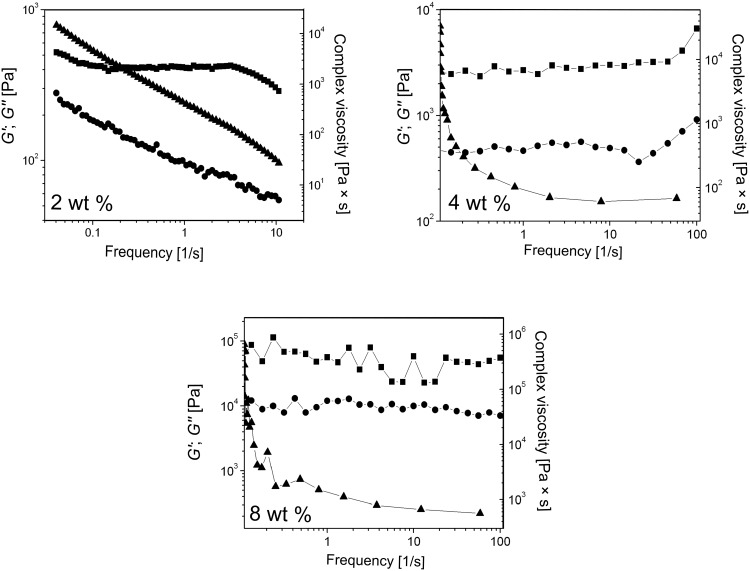
Frequency sweep tests and dynamic mechanical measurements of 2, 4, and 8 wt % **ODA-C**/silicone oil gels. *G*′ (■), *G*″ (●), and the complex viscosity, η* (▲; right axes) as a function of frequency, *ω*.

**Frequency sweep.** The behaviors of *G*′ and *G*″ as a function of the frequency for 2, 4, and 8 wt % **ODA-C**/silicone oil gels are shown in Figure 6. The oscillation amplitudes are in the linear viscoelastic regions as determined from experiments (Figure 5).

**Figure 7:**
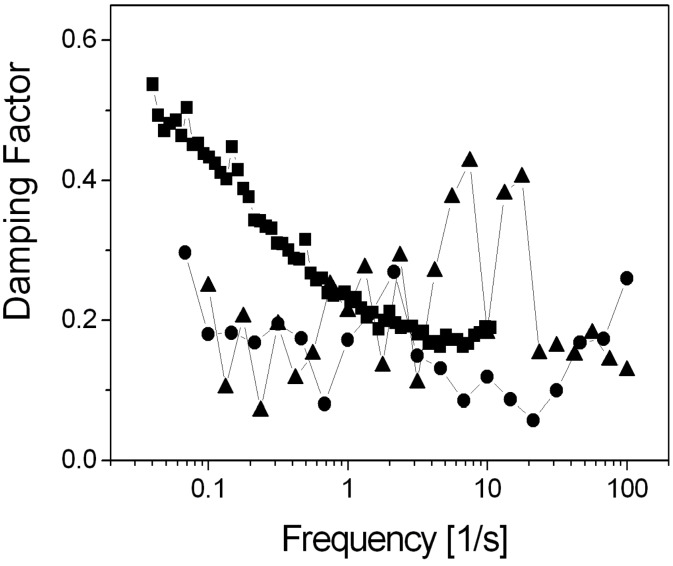
Damping factors of 2 (■), 4 (●) and 8 wt % (▲) gels at different frequencies.

The data are typical of a solid-like material: *G*′ remains larger than *G*″ over a very broad range of frequencies; there is no crossover between the loss and shear moduli within the range of the accessible frequencies. These results indicate that the physical junctions in the **SAFIN**s behave as permanent crosslinks (i.e., they have long lifetimes), typical of other self-assembled systems [18]. The assessment that the gel networks are solid-like is also supported by the fact that both *G*′ and *G*″ are more or less independent of frequency within the range investigated and increasing the **ODA-C** concentration leads to large increases in *G*′, *G*″, and η* (Figure 6). These conclusions are supported as well by the damping factors (DF = *G*″/*G*′) of the gels, which remain less than 1, as expected for a solid-like material, over the total frequency ranges examined (Figure 7).

Furthermore, because the *G*′ values for all the gel samples are nearly independent of the frequency of the applied perturbation, the average values of *G*′ can be considered equal to the intrinsic elastic shear modulus, *G* [19], which can be correlated with the entanglement density, *ρ*_e_, by the expression, *G = ρ*_e_*k*_B_*T* [20]. In these cases, the frequency of junction zones (i.e., where fibers of a SAFIN are joined [1]) may contribute to *ρ*_e_ as well. Thus, an increase in the elastic modulus *G*′ is indicative of an increase in the entanglement (or junction) density of the SAFIN fibers, even if the gross rheological behavior remains the same.

## Conclusion

Silicone solutions of the latent gelator, **ODA**, treated with CO_2_ gas produce viscoelastic materials that are true gels according to rheological criteria. The crystalline SAFINs are destroyed partially when exposed to excessive strain. It has been shown that very precise values of *T*_g_ are obtained by plotting the viscosities versus temperature and that these values should be more easily reproduced in other laboratories than those based on the falling drop method. The same data sets, when plotted in an Arrhenius fashion, yield activation energies for viscous flow within the gel and sol phases. As expected, the activation energies within the gel phases are at least one order of magnitude higher than those of the corresponding sol phases and, in some cases, more than 2 orders of magnitude higher. However, the rheological properties of the gels do not change linearly with latent LMOG concentration because the SAFIN structures must differ in subtle ways that cannot be understood by rheological measurements alone, but can be sensed acutely by them. Thus, rheology provides an extremely valuable tool to investigate the network structures of gels containing LMOGs.

## Experimental

Dry CO_2_ gas was bubbled through solutions of **ODA** (from Aldrich; distilled twice under vacuum and stored under a nitrogen atmosphere) in tetramethyltetraphenyltrisiloxane (Dow silicone oil 704 from Dow Chemical Company, Midland, MI) for several minutes to prepare 1-octadecylammonium 1-octadecylcarbamate (**ODA-C**) and the fibrillar structures of the gels derived from it [10]. Under a dry CO_2_ atmosphere and in sealed vials, the samples were heated to 75 °C for 5 min and slowly cooled (ca. 1 °C/min) to room temperature 3 times. This procedure yielded transparent sols that were cooled rapidly after the 4th heating by plunging the vials into a cold water bath at 2–3 °C. The resultant opaque, birefringent gels were then used for the rheological measurements.

Aliquots of the samples were heated again to 75 ± 2 °C and rapidly transferred onto the surface of the rheometer plate (initially at 10 °C above *T*_g_ [10] for 15 min). Then, the temperature of the plate was rapidly reduced (ca. 4 °C/min) to 25 °C; this annealing cycle was repeated 3 times prior to conducting the experiments at 25 °C. Virtually identical rheological data were obtained when aliquots of gel samples were transferred to the plate with a spatula at room temperature.

Rotational and oscillatory shear measurements were carried out with a plate–plate geometry (10 mm diameter) on a Paar Physica UDS 200 rheometer working in controlled shear stress. The dependencies of the storage modulus (*G*′) and the loss modulus (*G*″) on the oscillation frequency were obtained from the phase lag between the applied shear stress and the related flow and from the ratio between the amplitudes of the imposed oscillation and the response of the gel. The complex viscosity was also calculated from Equation 1.

[1]
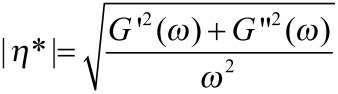


*G*′ and *G*″ were measured over a 0.001–100 s^−1^ frequency range. The values of the stress amplitude were examined by amplitude sweep tests in order to ensure that all measurements were performed within the linear viscoelastic region. All flow curve and oscillatory measurements were made at 25.0 ± 0.1 °C (Peltier temperature control system). Experiments to examine the viscosity at increasing temperatures were performed at a rate of 0.2 °C/min.

Optical micrographs were collected in the transmission mode by means of a Reichert Zetopan optical microscope equipped with an 11× objective and an 8× ocular using crossed Nicol polarizers.
